# Relationship between resident workload and self-perceived learning on inpatient medicine wards: a longitudinal study

**DOI:** 10.1186/1472-6920-6-35

**Published:** 2006-07-06

**Authors:** Elizabeth M Haney, Christina Nicolaidis, Alan Hunter, Benjamin KS Chan, Thomas G Cooney, Judith L Bowen

**Affiliations:** 1 Department of Medicine, Oregon Health & Science University, Portland, OR, USA; 2 Department of Medical Informatics and Clinical Epidemiology, Oregon Health & Science University, Portland, OR, USA; 3 Department of Public Health and Preventive Medicine, Oregon Health & Science University, Portland, OR, USA; 4 Department of Medicine, Veterans Affairs Medical Center, Portland, OR, USA

## Abstract

**Background:**

Despite recent residency workload and hour limitations, little research on the relationship between workload and learning has been done. We sought to define residents' perceptions of the optimal patient workload for learning, and to determine how certain variables contribute to those perceptions. Our hypothesis was that the relationship between perceived workload and learning has a maximum point (forming a parabolic curve): that either too many or too few patients results in sub-optimal learning.

**Methods:**

Residents on inpatient services at two academic teaching hospitals reported their team and individual patient censuses, and rated their perception of their learning; the patient acuity; case variety; and how challenged they felt. To estimate maximum learning scores, linear regression models with quadratic terms were fit on learning score.

**Results:**

Resident self-perceived learning correlated with higher acuity and greater heterogeneity of case variety. The equation of census versus learning score, adjusted for perception of acuity and case mix scores, showed a parabolic curve in some cases but not in others.

**Conclusion:**

These data suggest that perceived resident workload is complex, and impacted by additional variables including patient acuity and heterogeneity of case variety. Parabolic curves exist for interns with regard to overall census and for senior residents with regard to new admissions on long call days.

## Background

The resident work-hour and patient volume restrictions adopted by the Accreditation Council for Graduate Medical Education (ACGME) reflect an increasing awareness of the relationship between service and education for residents in training[1]. The rationale behind these requirements (workload caps) is to provide a safe and productive learning environment. The appropriate relationship between service and learning in medical education has been long-debated and remains controversial [2-4]. Service and learning are not completely distinct within medical education, because the practice of medicine itself is a service[5]. Medical education relies on learning in the context of providing clinical service to the patient. However, over-emphasis on clinical service, at the expense of other educational opportunities (discussion, reading, conferences or lectures) may be detrimental. Few data exist on the relationship between workload and learning to inform decisions about the balance between service and learning. Currently residency programs and residency governing bodies (such as the Internal Medicine Residency Review Committee) make such decisions by consensus. As yet, there is incomplete understanding of the components of resident work. Resident workload may reflect the number of patients cared for and/or the number of hours worked, but is also likely influenced by patient acuity (how sick the patients are) and case variety (how new and different the patients' illnesses are for the learner).

**Figure 1:**
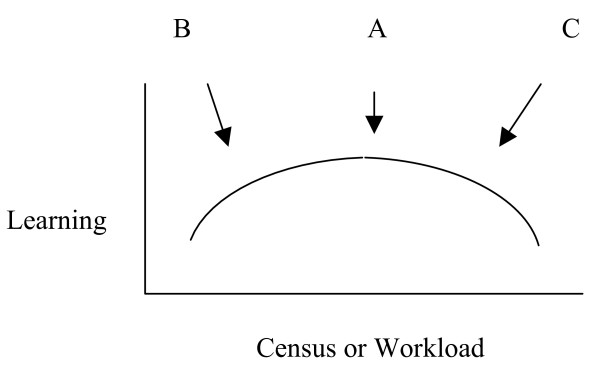
**Theoretical model of resident learning**. In a proposed theoretical model for resident learning, learning is optimal at a census or workload (Point A); where patient volume, acuity and variety (all contributing to workload) are adequate and appropriate for resident learning. Resident learning is less optimal when residents have either too few patients (inadequate workload, Point B) or too many patients (overwhelming workload, Point C).

In assessing our own program for compliance with ACGME census regulations, we recognized an opportunity to evaluate the relationship between workload and learning. Though we expected a linear relationship between residents' workload and their sense of feeling challenged, we felt that the relationship between workload and learning would less likely be linear. We hypothesized that resident workload and learning are related in a parabolic fashion (Figure 1). In this model, there is an optimal patient load, where resident learning is maximized (Point A). At either extreme, with either too few patients (Point B) or too many (Point C), resident learning is compromised. Ideally, residents will work in a range around the optimal patient number and workload. As knowledge and skills increase, this curve may shift rightward such that residents achieve optimal learning at a higher workload.

With this model in mind, our primary objective was to test the hypothesis that resident workload and learning are related in a parabolic fashion. We sought to define residents' perceptions of optimal patient workload for learning (Point A in Figure 1), and to determine how patient acuity and case variety contribute to resident workload beyond number of patients.

## Methods

### Population

We collected data from all residents (ie. in their 1^st^, 2^nd ^and 3^rd ^years of training: R1–R3) assigned to inpatient internal medicine services at two academic teaching hospitals, University and Veterans Affairs Medical Center (VAMC), from March 2001 through February 2002. Both institutions gave IRB approval for this study.

Our residency program has 90 residents. The resident call cycle at these hospitals is either 4 or 5 days long and the long call (LC) day refers to that day on which resident teams are the primary admitting team for the entire hospital. On the LC day, residents admit and work-up patients for 12–24 hours, often staying overnight at the hospital. Each university team has one intern and one resident; each VAMC team has two interns and one resident. Approximately 88% of the housestaff on the medicine wards are Internal Medicine (IM) residents; the others are psychiatry, neurology, emergency medicine or family practice housestaff. In our residency program, both R2's and R3's serve as senior residents on the ward teams.

### Survey instrument development and data collection

We created a survey instrument using an expert panel of 4 faculty members with administrative leadership roles in the residency program and 3 chief residents. We pilot tested the survey with residents over 1 month and made revisions to clarify items.

On weekdays for the middle two weeks of each month-long general medicine ward rotation over 12 months, residents completed questionnaires that assessed their individual patient censuses and the number of new admissions over the past 24 hours. Using a 5 point Likert scale, residents rated their perceptions of: 1) their learning for the day prior, ranging from *"less than optimal" *to *"ideal"*; 2) the acuity of their patient load, ranging from *"stable patients with straightforward problems" *to *"very sick patients with multiple problems and lots of diagnostic uncertainty"*; 3) the case variety of their patient load ranging from *"homogeneous patient problems, diagnoses you have seen before" *to *"heterogeneous patient problems, new and different diagnoses for you"*; and 4) how challenged they felt ranging from *"not challenged" *to *"overwhelmed"*. These operational definitions were included on the survey instrument (See Additional file 1).

The variables learning, acuity, case variety, and challenge refer to corresponding questions on the survey instrument. Census and new admissions refer to the number of patients reported by the idividual resident or intern. New admissions occur mainly on the LC day (in contrast to other days in the call cycle), so for analyses that looked at new admissions, we used only data from residents who had been on LC the preceding 24 hours. For all multivariate analyses, the dependent variable was resident self-reported learning. The independent variables were measures of patient volume (census and new admissions), and those that we hypothesized contribute in other ways to resident work (acuity and case variety).

### Statistical analysis

Responses were stratified by year of training. We analyzed data from R2s and R3s separately in preliminary analyses, but found that there were no statistical differences in the results. As there is also no clinical or educational difference in the work performed by R2s and R3s in our program, we present results with R2s and R3s combined.

We described the responses and used ANOVA to assess differences in means among training years. We performed bivariate correlations to assess for associations between the variables census or new admissions, challenge, acuity, case variety, learning. Additionally, we dichotomized the variables workload and learning to evaluate the relationship using chi-square statistics.

In order to estimate a relationship where learning increases as workload increases, but then declines at a certain unknown workload level, we included a quadratic term for workload in our regression models in addition to a linear term[6]. A statistically significant coefficient for the quadratic term indicates the relationship between learning and workload is parabolic, or U-shaped. Thus, to evaluate for parabolic relationships, multivariate linear regression models with quadratic terms were fit on the dependent variable learning score.

We adjusted the measures of patient volume (census or new admissions) for covariates that contribute to resident work (acuity and case variety) to achieve an overall measure of resident workload. We did not adjust for the variable challenge because we felt it was the causal pathway (learning is impacted by how challenged residents feel as a result of their census). Using the Beta-coefficients for the squared (*a*) and unsquared (*b*) pateint volume terms (ie census or new admissions), we calculated the maximum number of patients for each model using the formula *x*_*max*_*=-b/2a*. Statistical analyses were conducted using SPSS (Chicago, IL) and SAS (Carey, NC). All reported P values are 2-sided.

## Results

The overall response rate was 64%, yielding 1,422 surveys. Each resident responded to the questionnaire multiple times during a ward rotation. Approximately 37% of the surveys came from residents at the University and 63% from residents at the VAMC; this is consistent with the 4 team/8 residents structure at the unviersity and the 5 team/15 residents structure at the VAMC. R1's completed 56% of the surveys, R2's completed 26%, and R3's completed 18%, reflecting the distribution of residents on ward teams.

Table 1 shows survey responses according to resident year. Census, number of new admissions, and self-reported sense of challenge, acuity, case variety and degree of learning all varied by resident year. Census correlated with residents' self-preceived feelings of being more challenged (r = .544, p < .001). Case variety and acuity were also associated with residents feeling more challenged (r =. 41, p < .001 for case variety; r = .50, p < .001 for acuity).

For all resident years, learning score was statistically associated with the unadjusted census, but the correlation coefficient was relatively low and likely not clinically important (r = .098, p < .001). This was also true when learning and workload were dichotomized (see Additional file 2). Learning score was not associated with the number of LC new admissions (r = .082, p < .113). Learning correlated with acuity and case variety for all groups (r = .25, p < .001 for acuity; r = .33, p < .001 for case variety).

**Figure 2:**
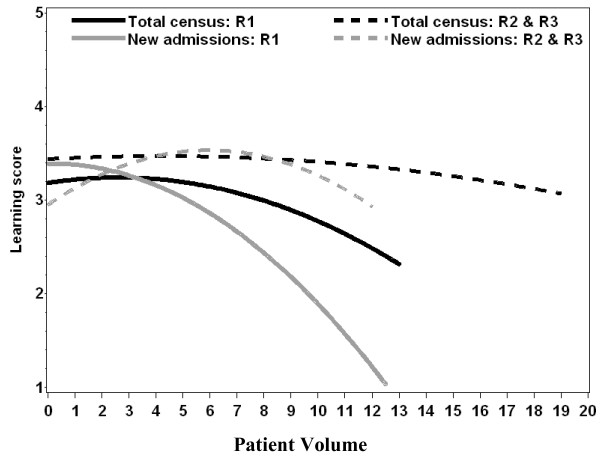
**Parabolic curves for learning vs. number of patients for R1's and R2/R3's**. Parabolic curves generated from multivariate models using quadratic equations demonstrate the relationships between learning and census or new admissions, adjusted for acuity and case variety for R1s as compared to R2s/R3s.

Table 2 lists regression coefficients for the multivariate models of learning and patient volume (as measured by either census or new admissions). Acuity was significant in the model for R1's relating learning to census; case variety was significant in all multivariate analyses. For interns (R1's), the quadratic term was statistically significant (P = 0.018) with perceived optimal learning occuring at a census of 3.1 patients. For senior residents, optimal learning would occur at a census of 6.6 patients, but the quadratic term was not significant (P = 0.144). Thus, interns, but not senior residents, have a parabolic line that describes the relationship between learning and census. There was no maximum number of new admissions identified for R1s. For senior residents, the optimal perceived learning occurred at 6.0 new admissions (P = 0.035). Figure 2 shows the parabolic curves for learning vs. patient volume (census and new admissions), adjusted for acuity and case variety.

## Discussion

We undertook a study to explore the relationship between workload and learning, to better understand the variables other than census that contribute to workload, and to see whether workload and learning would be related in a parabolic fashion after adjusting for variables contributing to workload. Our data demonstrate that residents report feeling more challenged as the number of patients they care for increases, as they see patients whose diagnoses are new to them (case variety) and who are sicker (acuity). These findings make intuitive sense. We found that patient acuity was independently associated with learning for interns caring for a census of patients, and that case variety was independently associated with learning for both interns and senior residents when admitting new patients and caring for them thereafter. The absence of a significant correlation between our measures of patient volume and learning suggests that the relationship is not linear, but is likely more complex. We attempted to fit a parabolic line to measures of patient volume and learning as one possible representation of these complex relationships.

With these data, we note that residents' self-perceived learning as it relates to patient volume adjusted for case variety and acuity fits a parabolic curve in some situations, but not others. Based on our knowledge of intern and resident workload, this may be understandable. For instance, the learning vs. census curves show a statistically significant maximum for the interns only. It may be that interns learn and are more challenged by the individual patients, so census plays a more important role in their learning. This would reflect the nature of an intern's work: writing orders, completing daily notes, admission and discharge dictations, and checking labs. It may also reflect interns' general lack of expertise, such that the daily tasks of caring for patients require slower analytic reasoning processes[7]. We found the optimal number of patients in their census, adjusted for acuity and case mix, to be 3.1. This number may not be clinically reasonable in and of itself, as much as the concept that for interns, there is a parabolic relationship between patient census and learning. In contrast, senior residents demonstrate statistically significant maximums for new admissions. This also reflects the workload for this group: residents generally feel challenged to think through new admissions, create and narrow a differential diagnosis, and direct initial management. If unfamiliar with diagnoses, their reasoning processes will more likely be analytic and more time consuming[7]. Thus, the curves that do have parabolic characteristics with significant maximums reflect where these two groups of learners spend most of their effort.

It may also be that the parabolic curve only becomes evident when a group is working both above and below their optimal workload during the period of observation. In other words, the relationship may be linear if data don't include a point that would be the maximum. For instance, even one new patient may challenge an intern. This could explain why the R1 curve for learning vs. new admissions is down-sloping and has no maximum. Likewise, if senior residents are theoretically only sufficiently challenged at a census of 15 patients, and they don't reach that census in our program, then the curve relating census to their learning would appear linear.

Our study has several important limitations. While we conducted the study at two separate teaching hospitals, the residents were all from one residency program. Although all surveys were anonymous, survey collectors anecdotally noted that they tended to collect fewer surveys from those residents who had busy services. Non-response bias toward the teams with larger censuses could explain why our data do not show a maximum for the senior residents; our data collection may not have captured their maximum. We surveyed all residents on our medicine teams, which included some non-IM housestaff. These residents may have different perceptions of learning or degree of challenge felt on medicine wards than do IM housestaff. While we collected data for an entire year, the collection period spans across two academic years. Thus, interns at the beginning of the study were in their second year of residency by the end of the study. Our own program underwent changes in the call cycle for inpatient medicine rotations during this study. We conducted analyses based on which trimester the data was collected and did not find that they significantly altered the results. We were unable to find a previously validated instrument that measured the concepts of perceived workload and learning so we developed our own instrument. This instrument has not been psychometrically tested and would have to be validated in another sample. Measures of acuity, case variety, and learning were based solely on resident perception. It is unclear how well residents' perceptions correlate with objective data. The surveys had no personal identifying information, so we are unable to account for clustering or adjust for demographic information. It is possible that the lack of adjustment for clustering may have led to false positive results. Similarly, we did not collect data on and thus were unable to adjust for, number of hours worked.

Resident responses may have been influenced by knowledge of the proposed ACGME caps on patient load. Residents are eager to please and to do what is expected of them. Indeed, they are taught that patient care comes above all else. Thus, they may mark optimal learning at the patient census and workload levels they feel are expected of them. They might not report being "overwhelmed" because they don't think they should be overwhelmed at a certain number of patients, or they may report being overwhelmed at the census that corresponds to the cap.

This study attempts to quantify inpatient learning as it relates to workload, and to explore factors other than census (case variety and acuity) that impact learning. Other studies have investigated time spent in learning activities after specific changes to rounds, and have evaluated the perceived value of educational activities by housestaff[8,9]. Our study begins the task of understanding the complex relationship between workload and learning, by defining workload not only as the number of patients, but also including patient acuity and case variety.

Further research is needed to confirm and improve upon these results. Electronic tracking of patient census, separate from resident perception surveys, would allow residents to be blinded to the nature of the study and report only on their learning. We recognize that training programs and hospitals differ. Our model and statistical method will need to be replicated at several institutions to test its ability to describe workload and learning in other settings. It is imperative that we consider ways to further understand the complex relationships that contribute to resident learning. With new regulations limiting not only patient numbers but also limiting work hours, we are challenged as educators to redesign resident training[4]. By understanding the components of resident learning more completely, we can be more certain of the impact of such changes on resident learning in complex clinical environments.

## Conclusion

Our data suggest that interactions between census and other variables such as case variety and patient acuity that contribute to resident workload are complex and make decisions about census cap limits more complex than currently portrayed. Components of resident workload other than just patient volume should be considered when making census cap decisions and when re-engineering graduate medical education. Additional research is needed to evaluate the components residents' workload and to optimize the learning environment.

## Abbreviations

ACGME – Accreditation Council for Graduate Medical Education

VAMC – Veteran's Affairs Medical Center

LC – long call

SC – short call

IM – Internal medicine

R1 – 1^st ^year resident

R2 – 2^nd ^year resident

R3 – 3^rd ^year resident

ANOVA – analysis of variance

## Competing interests

The author(s) declare that they have no competing interests.

## Authors' contributions

EH contributed to conception, design of study; collected and managed data; performed statistical analyses and interpreted the results; wrote and revised the manuscript.

CN contributed to statistical analyses and interpretation of results; revised draft manuscript and approved final manuscript.

AH contributed to conception, design and interpretation of data; contributed to data analysis; revised draft manuscript and approved final manuscript.

BC performed statistical analyses and interpreted results; revised draft manuscript and approved final manuscript.

TC contributed to conception, design and interpretation of data; revised draft manuscript; approved final manuscript

JB contributed to conception, design and interpretation of data; revised draft manuscript and approved final manuscript

## Pre-publication history

The pre-publication history for this paper can be accessed here:



## Supplementary Material

Additional File 1 Resident learning survey, survey instrument administered to residents. Click here for file

Additional File 2 2 × 2 tables and chi-square statistics for dichotomized learning and volume measure variables, data for learning and workload using dichotomized variables for R1s and R2/3s. Click here for file

## Figures and Tables

**Table 1:** Survey Responses According to Resident Year of Training

	**R1**	**R2/R3**
N	793 (59%)	626 (44%)
Census	4.7 (SD 2.3)	7.5 (SD 3.5)
New Admissions	3.4 (SD 1.9)	5.2 (SD 2.7)
Acuity	3.1 (SD 0.91)	3.2 (SD 0.89)
Case Variety	3.3 (SD 1.0)	3.5 (SD 0.88)
Learning	3.1 (SD 0.86)	3.3 (SD 0.87)

All comparisons between R1s and R2/R3s are significant at p < 0.001.

**Table 2:** Parameter coefficients for learning vs. patient volume multivariate models

	**Relationship Between Census and Learning**				**Relationship Between New Admissions and Learning**			
	
	**R1's**		**R2's and R3's**		**R1's**		**R2's and R3's**	
	
	Beta coef.	p-value	Beta coef.	p-value	Beta coef.	p-value	Beta coef.	p-value
**Acuity**	.108	.006	.0605	.175	.0834	.279	.114	.191
**Case Variety**	.267	<.001	.264	<.001	.355	<.001	.192	.038
**Census squared**	-.0095	.018	-.0030	.144				
**Census**	.0592	.194	.0393	.277				
**Optimal census**	3.1		6.6					
**New admits squared**					-.0151	.220	-.0146	.045
**New admits**					.0059	.955	.190	.022
**Optimal new admits**					None identified		6.5	
