# Supplementary material for: Relationship between resident workload and self-perceived learning on inpatient medicine wards: a longitudinal study
Source: BMC Med Educ. 2006 Jul 6;6:35. doi: 10.1186/1472-6920-6-35 (PMC1550230; doi:10.1186/1472-6920-6-35)
Supplement: Additional File 1 — Resident learning survey, survey instrument administered to residents. [file 1472-6920-6-35-S1.doc]

# Additional file 1: Resident learning survey

Date: _____________ Level of training: R1 R2 R3

Current ward service _______________ Hospital: VA University

Residency: IM FP Psych Neuro

What day of the call cycle were you on **yesterday**? LC PC NFA SC Rounds Off

Did you have clinic **yesterday**? Yes No

Total team census **this morning**: __________ **R1's only**: How many patients are you caring for **this morning**? ___________

How many **new admissions** did you evaluate between 8am yesterday and 7am today? ___________

(include bounce backs, NFA, and inter-team transfers)

1. In terms of the **total number of patients** you cared for yesterday (8am to 8am), how do you feel?

Not challenged 1--------2--------3--------4--------5 Overwhelmed

Why? _______________________________________________________________

2. In terms of the patients you cared for yesterday, how do you rate the **average acuity**?

Not acute Very acute, sick patients

Stable, standard problems 1--------2--------3--------4--------5 Multiple problems

Straightforward Lots of diagnostic uncertainty

Comments? _________________________________________________________

3. In terms of the patients you cared for yesterday, how variable was the case mix?

Homogeneous Heterogeneous

(Diagnoses you have 1--------2--------3--------4--------5 (new and different

cared for before) diagnoses for you)

Comments? _________________________________________________________

4. How would you classify your learning over the past 24 hours?

Less than optimal 1--------2--------3--------4--------5 Ideal

Why? ____________________________________________________________________

1. **How important** were each of the following in your learning yesterday?

**Not at all Important** **Extremely Important**

Learning from faculty attendings/consultants 1--------2--------3--------4--------5

Learning from peers (other residents, interns) 1--------2--------3--------4--------5

Looking things up/self directed reading 1--------2--------3--------4--------5

Attending conferences 1--------2--------3--------4--------5

Patient care (learning by doing) 1--------2--------3--------4--------5
