# Supplementary material for: Relationship between resident workload and self-perceived learning on inpatient medicine wards: a longitudinal study
Source: BMC Med Educ. 2006 Jul 6;6:35. doi: 10.1186/1472-6920-6-35 (PMC1550230; doi:10.1186/1472-6920-6-35)
Supplement: Additional File 2 — 2 × 2 tables and chi-square statistics for dichotomized learning and volume measure variables, data for learning and workload using dichotomized variables for R1s and R2/3s. [file 1472-6920-6-35-S2.doc]

**Additional file 2: 2x2 tables and chi-square statistics for dichotomized learning and volume measure variables.**

R1

|  | Good Learning | Poor Learning |
| --- | --- | --- |
| High Workload (census) | 200 (76%) | 62 (24%) |
| Low Workload (census) | 378 (77%) | 115 (23%) |

Chi-square: 0.01, P=0.92

R2/3

|  | Good Learning | Poor Learning |
| --- | --- | --- |
| High Workload (census) | 95 (80%) | 23 (19%) |
| Low Workload (census) | 408 (83%) | 84 (17%) |

Chi-square: 0.38, P=0.5

R1

|  | Good Learning | Poor Learning |
| --- | --- | --- |
| High Workload (new admits) | 19 (61%) | 12 (39%) |
| Low Workload (new admits) | 550 (77%) | 164 (23%) |

Chi-square: 4.1, P=0.04

R2/3

|  | Good Learning | Poor Learning |
| --- | --- | --- |
| High Workload (new admits) | 4 (57%) | 3 (43%) |
| Low Workload (new admits) | 468 (83%) | 93 (17%) |

Chi-square: 3.4, P=0.07

Good learning = rated as 3, 4 or 5 by the resident

Poor learning = rated as 1 or 2 by the resident

High Workload (census) = census greater than 5 for R1, census greater than 10 for R2/3

Low Workload (census) = census of 5 or fewer for R1, census of 10 or fewer for R2/3

High Workload (new admits) = new admissions > 5 for R1, new admissions >10 for R2/3

Low Workload (new admits) = new admissions of 5 or fewer for R1, new admissions of 10 or fewer for R2/3
